# A “Behind-the-Scenes” Look at Interprofessional Care Coordination: How Person-Centered Care in Safety-Net Health System Complex Care Clinics Produce Better Outcomes

**DOI:** 10.5334/ijic.4734

**Published:** 2020-04-28

**Authors:** E. Marshall Brooks, Jodi M. Winship, Anton J. Kuzel

**Affiliations:** 1 Virginia Commonwealth University, Department of Family Medicine and Population Health, Richmond, VA, US; 2 Virginia Commonwealth University, Department of Occupational Therapy, Richmond, VA, US

**Keywords:** Complex Care Clinics, integrated care, care coordination, interprofessional teams, high utilizers, person-centred care

## Abstract

**Introduction:**

While the effectiveness of team-based care and wrap-around services for high utilizers is clear, how complex care clinics deliver effective, person-centered care to these vulnerable populations is not well understood. This paper describes how interactions among interprofessional team members enabled individualized, rapid responses to the complex needs of vulnerable patients at the Virginia Commonwealth University Health System’s Complex Care Clinic.

**Methods:**

Researchers attended twenty weekly care coordination meetings, audio-recorded the proceedings, and wrote brief observational field notes. Researchers also qualitatively interviewed ten clinic team members. Emergent coding based on grounded theory and a consensus process were used to identify and describe key themes.

**Results:**

Analysis resulted in three themes that evidence the structures, processes, and interactions which contributed to the ability to provide person-centred care: team-based communication strategies, interprofessional problem-solving, and personalized patient engagement efforts.

**Conclusion:**

Our study suggests that in care coordination meetings team members were able to strategize, brainstorm, and reflect on how to better care for patients. Specifically, flexible team leadership opened an inter-disciplinary communicative space to foster conversations, which revealed connections between the physical, and socio-emotional components of patients’ lives and hidden factors undermining progress, while proactive strategies prevented patient’s rapid deterioration and unnecessary use of inappropriate health services.

## Introduction

High utilizers of health services pose a unique problem to hospital systems. Less than 1% of patients account for 21% of U.S. healthcare expenditures, with the bulk of resources spent on hospital costs [1]. Creating high quality and efficient models of care delivery for high utilizers can help achieve the Triple Aim of better care, smarter spending and healthier people [2,3,4]. Yet the needs of so-called “high utilizers” with multiple preventable Emergency Department visits and inpatient hospitalizations are complex [5,6]. They often have an array of physical, social and behavioural health needs and complications that traditional clinic models are unable to address [7,8,9,10].

Extending beyond the mere co-occurrence of clinical comorbidities, “patient complexity” refers to a constellation of biopsychosocial issues, including, unstable employment and housing, low health literacy, educational deficits, social isolation, and mental illness and substance use disorder [1,5,10]. Following Peek, Baird and Coleman [9], patient complexity consists of a set of “person-specific factors” that “interfere with the delivery of usual care and decision-making for whatever conditions the patient has.” In the short term, such factors can lead to patient difficulties with scheduling, attending clinic appointments, self-managing medications, and adhering to treatment plans. In the long term, these also lead to increased Emergency Department use and increased inpatient hospital admissions [6].

Complex care clinics – interprofessional clinical teams providing enhanced care coordination and case management to a health systems’ highest utilizers – are a novel strategy for addressing these person-specific challenges [11]. In addition to coordinating among specialists, tracking transitions in care, and providing medication management, complex care clinics provide individualized, person-cantered care responsive to patients’ complex lives, including a constellation of chronic and acute physical illnesses as well as social and interpersonal dimensions of their personal lives [12]. Published accounts regarding the effectiveness of team-based care and wrap-around services for high utilizers have shown reduced emergency room visits and hospital admissions, improved clinical outcomes, decreased symptoms, improved adherence to treatment, and lowered costs of care [13,14,15]. But, *how* Complex Care Clinics go about delivering effective, person-centred care is not well understood [16].

Previous evaluations of care-coordination within ambulatory settings have demonstrated mixed results [17]. Failed attempts at successful care-coordination have been partially attributed to the lack, or poor implementation, of critical program features, including: frequent in-person patient contact, routine communication among providers, delivery of evidence-based patient education, strong medication management, and timely, comprehensive transitional care after hospitalizations [18]. Previous research similarly identifies professional, organizational, and interpersonal factors that contribute to successful interprofessional collaborations, including the need for mutual trust and respect, timely communication, and strong leadership [19]. While such a list of critical factors is helpful, existing literature focuses on general recommendations to include these core features of interprofessional care planning yet leaves unspecified what the “behind the scenes” activity of providing such services looks like. Specifically, this research sought to answer the following questions: How do interprofessional teams organize themselves to holistically address medical and social complexity? How is an emphasis on person-centred care established and maintained? And how do regular care coordination meetings facilitate positive health outcomes?

To address these questions, this paper describes how one complex care clinic working in a safety-net setting provided holistic, person-centred care to a patient population with complex biological, social, and behavioural needs and challenges. We focus on weekly clinic team meetings as a site through which attentiveness to patient complexity emerged and was operationalized. Specifically, we describe how regular team meetings opened a multi-disciplinary communicative space in which the clinic creatively tailored its care to each patient’s unique needs and challenges. While previous literature has discussed the organizational structures and processes through which care coordination unfolds, a qualitative description of team meetings as a key component of this has been missing.

### Setting: The Virginia Commonwealth University Health System Complex Care Clinic

Virginia Commonwealth University (VCU) Health System’s Complex Care Clinic was started in 2011 to meet the needs of complex uninsured and Medicaid patients with the highest costs and utilization of Emergency Department and inpatient services in the VCU Health System, a safety-net health system based in Richmond, Virginia [20]. The team consists of two internists, a nurse practitioner, two nurse case managers, three registered nurses, two post-doctoral psychology trainees, a social worker, a pharmacist, and three community outreach workers. Together, they coordinate care for approximately 700 patients with multiple chronic physical conditions as well as behavioural health and social issues (Table 1). While many team-based care approaches involve a coordinated approach with delegation of tasks, the Complex Care Clinic uses an interprofessional team-based approach with each contributing a specific aspect of care through direct interaction with the patient, often during the same visit. Although each clinician manages a specific aspect of care (e.g., the physician diagnoses and prescribes medications, the psychologists addresses mental health issues, the social worker finds community resources, etc.), the team works collaboratively to address patient needs. Their collaborative visits focus on whole person care, drawing needed expertise into a single location, engaging patients in their care, addressing patient barriers, and connecting them with appropriate services. In addition to patient visits, the team meets weekly to organize care and establish priorities. In other studies, this type of team-based approach to care has proven to be successful in improving the quality of care for patients and lowering costs [21,22]. Such successes are reflected in the VCU Health System Complex Care Clinic’s outcomes in their first year of operation, including: 44% decline in inpatient hospitalizations, 38% decrease in emergency department use, and 49% reduction in total hospital costs. They achieved a total of $4 million total cost savings for 365 patients that first year, with an average annual cost savings per patient of $10,769 [23].

**Table 1:** Demographic, socioeconomic and clinical characteristics of FY15 Complex Care Clinic patients.

	CCC patients (N = 806)

Median Age at start of FY	52.0
**Race and ethnicity**	**N (%)**

Non-Hispanic Black or African American	529 (65.6)
Non-Hispanic White	228 (28.3)
Non-Hispanic Other	35 (4.3)
Hispanic, any race	14 (1.7)
Female	379 (47.0)
**Payer at last visit**	

Commercial	40 (4.9)
Medicaid	177 (21.8)
Medicare	160 (19.8)
Uninsured	361 (44.6)
Other or unknown	71 (8.8)
Unemployed	666 (82.6)
Live in area with greater than average poverty rate†	407 (50.4)
Median income	$33,647
**Top 5 most frequent diagnoses**	**N (%)**

Diabetes	426 (52.8)
Mental illness	400 (49.6)
COPD	333 (41.3)
Congestive heart failure	255 (31.6)
Drug or alcohol abuse	190 (23.5)
3 or more comorbid conditions	496 (61.5)

## Methods

We conducted an exploratory observational case study [24] with clinicians in an urban, academic complex care clinic from October 2014–May 2015. Qualitative data were collected from care coordination meetings and interviews with clinical team members. Data were analysed using conventional content analysis methods. The interviews, along with clinic observations and other qualitative data, were collected as part of a separate study examining best practices in care coordination. The original study received approval from VCU’s Institutional Review Board, and written consent was obtained from all study participants.

### Data collection and analysis

Two types of qualitative data were collected across the eight-month study period. First, researchers attended twenty weekly care-coordination meetings that were audio-recorded and transcribed across a four-month time frame. The researcher attending the team meetings did not sit at the table with the team, rather they remained in the back as an observer, managing the logistics of the recording equipment and taking notes to assist in the subsequent transcription of the meetings.

Second, researchers audio-recorded and transcribed semi-structured interviews with ten Complex Care Clinic team members, each thirty to forty-five minutes in length. Clinician interviews were conducted individually in private spaces in or near the clinic. All full time Complex Care Clinic clinicians were interviewed (N = 10), excluding one physician who was on leave for the duration of the study (Table 2). Interview guides included questions about the clinic process and procedures, challenges and successes, patient engagement, and team-based care. The interviews were conducted by a PhD-trained medical anthropologist, a research associate in the Department of Family Medicine & Population Health, or an occupational therapy student. No one on the research team was affiliated with the Complex Care Clinic. All interviewers were trained on the qualitative research process and how to conduct semi-structured interviews. The interviews were audio recorded and transcribed verbatim by professional transcriptionists.

**Table 2:** Clinician sample.

Title	Number of Participants

Clinical Nurse	1
Clinical Psychologist	2
Nurse Practitioner	1
Pharmacist	1
Physician	2
Clinic Director	1
Nurse Case Manager	1
Social Worker	1

Both the team meeting and interview transcripts were uploaded into the qualitative data analysis software, Atlas.ti, and concurrently coded using both template-based and emergent coding techniques to thematically analyse our qualitative data [25,26,27]. First, a subset of the transcripts (both clinician interviews and team meetings) were read and coded by a three-member coding team, including a medical anthropologist, a primary care clinician, and a research associate, using an a priori codebook with codes derived from the interview guide (i.e. organizational structures, care-coordination, and interprofessional interactions.) Using constant comparison [28], the coding team met regularly to ensure consistent application of codes and to identify and discuss emerging analytical patterns in the data until agreement on the number of codes and the definition for each code was achieved [29]. Once the team reached saturation regarding identified codes, the remainder of the transcripts were coded independently by two coding team members, codes were compared and any discrepancies in coding were discussed until consensus was found. Finally, the codes were analysed as a group to develop emergent patterns and themes present in the data [30].

## Results

Analysis of the team meetings and interviews resulted in the identification of three themes: team-based communication strategies, interprofessional problem-solving, and personalized patient engagement efforts, which contributed to the Complex Care Clinic’s ability to provide person-centred care. Presentation of results below is organized around these themes.

### Team-Based Communication: Flexible Team Leadership, Goal Focused Conversation, and Self-Reflexive Dialogue

Care-coordination meetings were ostensibly led by the nurse practitioner, who was responsible for shepherding the conversation through the clinic’s entire list of priority patients for the week. But team discussions evidenced little pre-determined authority structure and instead relied on an overall sense of shared-responsibility for patient care. During meetings, each Complex Care Clinic team member provided an update on each patient based on their personal interactions. This person-centred form of holding team discussions often revealed unidentified problems or challenges with patients and provided an opportunity for team members to collectively strategize their approach to care. For example, here is the nurse practitioner providing a summary at the end of a conversation about how the nurse and community outreach worker will coordinate patient education.

“[Nurse] is going to work with [outreach worker] in terms of being the point person, but also educating her on the foundational information. So, [nurse] is going to teach [outreach worker] the same kinds of stuff that she would teach a patient so that she has that same understanding with those teaching tools, imparting what [nurse] would hope that patient would learn, and reinforcing it with that patient.” (Nurse Practitioner)

Because complex patients often have multiple chronic and acute physical illnesses as well as social and interpersonal challenges, the Complex Care Clinic team used a form of flexible team leadership where the goals and priorities of patient care were diffused across the team rather than centralized in a singular “team leader,” or dominated by purely biomedical concerns. This was evidenced in the generally open dialogue that transpired around issues of greatest importance to individual patients–whether about adjusting medications or providing assistance securing unemployment benefits–and in the way those with the most knowledge about or experience with the patient–regardless of professional title–set the priorities and guided the person-centred discussion. In particular, frequent contributions from the social worker and community outreach worker allowed the team to adjunct strictly biomedical care plans with comprehensive approaches that addressed the full scope of patients’ social, behavioural and psychological health issues. For example, here is the social worker leading discussion around a man with complex psycho-social needs.

“He has intermittent homelessness, in large part due to his substance abuse issues. He has been always coming to the Emergency Department because he does not take his anti-seizure medication. And that’s been in part because he has poor short term and long-term memory. So, we’re trying to find out how he can deal with his substance abuse. And working with [detox centre], for him to get an extended inpatient stay, which will hopefully clean him up and get his memory to improve a little bit…. There’s a lot of agencies working for him to help him overcome his hurdles. But the thing is, one of his triggers is that when he gets the money from his disability, he goes straight to purchase drugs. So that’s another built in hurdle for him to overcome. So, I’m working with these other people to see how we can all tie in what we do for him that will give him a plan of sobriety.” (Social Worker)

Although frequently dominated by patient complexities, the team also regularly incorporated patient’s self-articulated treatment goals and preferences, recorded in the patient’s chart, into team discussions. Reflecting on this practice during a team meeting, one of the physicians recounted a personal example of the unexpected benefits of incorporating a more holistic awareness of patient goals and priorities into clinical decision-making.

“She went to dialysis three days: Monday, Wednesday, Friday. Then over a couple years, she started getting hypoxic and short of breath and looking like crap, went on oxygen. When I started doing the disability exams, I called her to see if she wanted to do peritoneal. It allowed her to travel and she was better when she went to the peritoneal. All of these issues that had started coming up in her resolved when she started doing it herself. And then more importantly, it gave her the freedom – she had family in Jersey – she was able to go to Jersey and visit folk and do all other kind of activities.” (Physician)

Additionally, voicing patients’ goals provided an added dimension of humanity to what could risk becoming a detached and clinical discussion of patients’ medical problems. This feature of the meetings seemed to bolster team members’ empathy for the struggles patients faced while trying to take care of their health needs. For example, one nurse care manager voiced annoyance with a patient who was attending dialysis appointments but frequently missing Complex Care Clinic visits. After listening to her frustrations, the social worker provided a more in-depth explanation of the man’s actions, including a description of the man’s priorities and the daily struggle he faced running his private business. Afterwards, the team transitioned from lamenting some patients’ lack of engagement, to constructive and empathetic problem solving. They ultimately decided to find a primary care provider with whom they could partner to provide care closer to the patient’s home and work.

Finally, during care-coordination meetings the team evidenced an ability to engage in constructive, self-reflexive dialogue about how clinic operations could be better adapted to meet the diverse needs of their patients. For example, in the following excerpt the nurse case manager is reflecting on challenges with a new patient who recently began missing appointments. Here we see a holistic awareness of how issues of clinic operations, person-centred principles, and patient engagement efforts intricately overlap.

“I think she’s had a big health scare, but I think that we could easily get her disappointed, or kind of disillusioned, if she has to wait a long time for her appointments, and so if we find that that’s a concern, we need to think if there are other options for her. You know, she’s motivated, she wants to get help. She understands that the visits might be long. But our goal should be to have a clinic that provides team-based care to people with different insurers. We have to realize that many of our patients are chronically ill and trying to manage their schedule and employment.” (Nurse Manager)

### Interprofessional Problem-Solving: Identifying Strategies to Provide Holistic Patient Care and Appropriate Use of Healthcare Services

Team members reported that the clinic’s interprofessional model of care allowed patients to benefit from access to multiple providers during an appointment, that not only met their medical needs, but behavioural, emotional, housing, and community support needs as well:

“We are multidisciplinary depending on the days, we’ll usually check and huddle in the morning to see if they’re [patients] going to be seeing other providers or does social work need to check in …Sometimes Psych gets here and following up on their anxiety and depression or maybe smoking cessation … Pharmacy will do diabetes, they’ll do general medication management, hypertension. Maybe suggest nutrition consult for our obese patients who are …trying to get cheap healthy foods.” (Clinician)

But changes to circumstances and health can happen quickly with complex patients and thus require heightened attention and vigilance. To achieve this, in care-coordination meetings the Complex Care Clinic team engaged in a transdisciplinary form of problem-solving focused on mitigating the various, and at times unidentified, factors that undermine patients’ health. In particular, the interprofessional nature of the clinic allowed the team to talk with one another to jointly problem solve emergent concerns and challenges as well as learn from one another about how to better engage patients.

Nurse Practitioner: “[Patient] keeps bouncing in and out. He’s in the Emergency Room. I’m not sure if he’s still down there. He hasn’t been able to be reengaged with our clinic. Not sure really what’s going on. I don’t even know if we know how to really get in touch with him…”

Physician: “He likes pain medicine…”

Nurse Practitioner: “Yeah, but I think they’re kind of getting hip to that in the Emergency Room. So, [social worker], see if maybe you can see if you can touch base with him. ‘Cause he missed his follow-up last go around.”

Typically focused on patients identified as lacking motivation to adhere to treatment plans or at risk of disengaging from the clinic, through such conversations the team realized connections among the physical, emotional, and social aspects of patients’ lives and the individually unique barriers and facilitators to care. In doing so, these conversations pro-actively reflected upon the social and emotional dimensions of health and illness and leveraged this into a more holistic understanding of peoples’ challenges and a more comprehensive approach to patient care.

“One of the patients I had was [patient name]. And in talking to her – she recently got readmitted, yesterday, for gallstones – she said a way that we can help her is with her financial problems, which she says comes from her leg injury and where she may have to go in and get more work done on her foot.” That has created a strain in her relationship with her live-in boyfriend who works a minimum wage job. As a result, she becomes despondent and tends to not want to do self-care, in terms of following the recommendations of the doctor and her therapist. So, she spirals down, and that’s when she has suicidal ideation because she figures, “well, my foot is not getting well and my boyfriend is tapped out as far as how much he can earn because he do a lot of overtime.”

So, I said, “What can we do?” And she said in that respect, she’d like more contact. And this is something I’d like to table with the outreach worker, who I’m going to have to work real close with. I tell them they have to be the eyes and ears of the staff when they go into the home, to see what’s going on with the patient, to see if they have any needs that they’re not really saying for fear, for shame, for embarrassment, or whatever. [Case manager] told me how little they have in the home, because they’re covering their major bills, which is rent and utilities. That way we can be more proactive in helping her. With all her issues, we really need to find out what’s going on in the home. “Do they have their daily needs being met? Then I can assess resources that could help them in some way.” (Social Worker)

Similarly, team members frequently discussed the ways in which friends and family can complicate patient’s visits. In one meeting a physician noted that one of her patients appeared aloof and disengaged during visits. She voiced a concern with how the patient’s mother, who always accompanied her adult son to appointments, seemed to affect the patient’s participation in his own care. “I’ve never asked her to leave…but thought I’d bring it up and see if anyone has suggestions,” the physician asked the team. Others had also in fact noticed this trend. “I think that his mother is over-nurturing” the social worker replied, “he’s guarded around his mother.” The social worker then suggested that, in his experience, the man was “more expressive” and able to articulate personal goals for his health and life when engage done-on-one. Given this new information, the team’s discussion turned to identifying ways of occupying the mother during appointments so that the physician and patient could talk one-on-one.

This strategy also included identifying the social situations and stressors that previously precipitated changes in a patient’s health and devising interventions that extended beyond traditional biomedical treatment options. For example, one particularly complex patient had a stroke and was sent to the Emergency Department after suffering workplace abuse. He told the social worker that the incident made his blood pressure “go through the roof” before blacking out. His case was further complicated by his dependence on staying at the job where the abuse occurred in order to maintain secure housing. During the care coordination meeting, the social worker was able to share this information with the group and to ask the patient’s physician to write a letter enabling light-duty work, away from the abusive co-workers, until he had time to recover or find alternate housing.

While care-coordination meetings increased the team’s ability to provide patients with services when and where needed, it also was an effective means of preventing the overuse of services. Greater knowledge of patients’ life circumstances – their assets as well as their barriers – empowered the team to make more effective use of services, and decide when less intensive, and often less expensive, options were more appropriate. In particular, the team often discussed patients who, when approaching discharge from the hospital, claimed to be unable to “take care of [them]selves” at home and were requesting inappropriate forms of higher level care. This often included a critical discussion of the patient’s home life and what other problems they were trying to avoid by requesting to stay in the hospital. For example, one female patient was about to be discharged from a residential rehab facility where she was recovering from an abscess. Although the wound had healed, she continued to complain that she was experiencing pain, could not provide for herself at home, and would inevitably burden her family. At the next team meeting, team members constructed a multi-dimensional understanding of the woman’s situation: the physician confirmed that the brace prescribed was no longer clinically necessary; the care manager described the patient’s observed capacity to perform the basic and instrumental activities of daily living; and the social worker provided insight into the patient’s home life that contributed to her fear of being discharged. As a result, the team discussed alternative options, including offering physical and occupational therapy at home or in a shelter, so that the woman could receive care without maintaining residency in the treatment facility.

### Personalizing Patient-Engagement: Building Relationships of Trust to Improve Person-Centered Care

Many high utilizer patients have experienced hardship or trauma making it difficult to build long-term, trusting relationships, especially with health care providers [30]. Complex Care Clinic members were aware of this problem but also felt that good patient-clinician relationships were a strong predictor of sustained patient engagement. Thus, they actively sought to develop such relationships in order to foster patients’ sense of “investment” and feeling “connected” to the clinic. Complex Care Clinic team members believed this resulted in increased patient buy-in, better treatment adherence, consistent appointment attendance, and greater trust in providers.

In care-coordination meetings, team members discussed patients struggling to stay engaged with the clinic who could benefit from a more “personal touch.” For example, the Complex Care Clinic team once identified a newly enrolled man who, embarrassed about his obesity, expressed hesitation to visit the clinic. After a brief discussion, the group decided that the community outreach worker with whom the patient seemed fond of should “develop a relationship” with the patient to make him feel more comfortable and ultimately “draw him in” to the clinic.

Nurse: “So, does that mean that [outreach worker] is charged with making sure compliance with meds and attending appointments?”

Outreach worker: “Yeah, ‘cause he comes back in the next month, I believe, to see [pharmacist].”

Physician: “So he’s one of those people that we can’t wait four weeks…”

Outreach worker: “Yeah, no.”

Physician: “…to touch out to him. He’s one of those people that need– you know, he probably needs a little handholding once a week or even twice a week to say, “Hey Mr. [patient name], how are things going? You taking your meds? Remember, yadda yadda yadda.” And to kind of look in IDX [scheduling system] to see when other appointments are there. Because he really has to demonstrate that he can adhere to regimen. So, follow-up calls and he’s just that simple, that he needs to have some handholding.”

Nurse: “I’d go twice a week for sure.”

Outreach worker: “OK.”

Nurse: “And then you might have to ratchet up if he’s sounding like he’s not taking his meds.”

To achieve such person-centred relationships with patients, the interprofessional team capitalized on the diversity of its members, each with different training and perspectives who, ultimately, were able to relate to patients differently.

“You know, like some patients don’t like seeing me, maybe because of my status…and I think sometimes just because they don’t find any benefit. There are other patients who really don’t like going to see other providers for similar reasons; either because they don’t see benefit, or they have a negative interaction. So, I think the team’s helpful in that way, ‘cause then there’s other personalities and other faces to communicate and engage the patient if one person can’t.’” (Clinician)

Similarly, the team also attempted to cultivate intentional relationships with patients who were particularly resistant to full engagement with the Complex Care Clinic. For example, one patient often pleaded with team members when challenged about her lack of personal responsibility in showing up for appointments on-time, keeping up regular communication with clinic staff, or adhering to treatment plans. Team members found it difficult to balance having respect for, and being supportive of, this patient’s challenges, while also pushing her to take personal responsibility. Discussing this during a coordination meeting allowed the team to match the patient with the team member most able to balance respect for the patient’s emotional needs while offering firm guidance and candidly talking about things the patient may not want to hear.

Many Complex Care Clinic patients had not previously received regular primary care or had not received care successfully. As a result, they were not familiar with the usual expectations, such as attending appointments on-time, scheduling and participating in clinic visits, or appropriately interacting with clinic staff. Complex Care Clinic team members routinely met with patients during an on boarding process to discuss the expectations and to ask them to sign a patient contract agreeing to the terms of enrolment. Sometimes this one-time instruction was not enough to change the patient’s habits. Coordination meetings enabled team members to identify and discuss those patients in need of further instruction. For example, during one meeting a team-member referred to a patient who had recently visited the clinic while intoxicated, continued to drink alcohol in the waiting room, and was reportedly “smelling foul.” Seeing an opportunity to intervene, the social worker volunteered to initiate “a long talk about appropriateness… and expectations.” To further bolster his efforts, the team assigned an outreach worker to “adopt” the man and reinforce these expectations during her regular pre- and post-visit phone calls with the patient.

Although often effective, the team also recognized that not every patient wants such a relationship and the team cannot always be the one to choose with whom patients will feel connected. For example, some patients were hostile with providers the clinic thought might make a best match and instead became attached to another Complex Care Clinic provider tangential to their care. The team frequently used this to their advantage and tailored their patient outreach efforts to capitalize on that affinity.

## Discussion

Clinicians and patients have similarly recognized the benefits of interprofessional care coordination in the complex care clinic, including effectively addressing the range of needs affecting the health of the patients through using the variety of resources on the team and team-based problem solving [31]. Our findings support other studies highlighting the benefits of interprofessional teamwork in primary care [19,32,33,34]. However, our findings add an analysis of care-coordination conversations during team meetings to the literature on case management and Complex Care Clinics by describing the “behind-the-scenes” operations of delivering person-centred care [35,36]. Our study is the first that we know of that identifies how interactions and communication between interprofessional team members enables an individualized, holistic and timely response to the complex needs of vulnerable patients, unlike previous studies that have focused solely on the organizational components of effective case management [37,38].

Our findings further contribute to a growing body of literature that suggests communication and interaction among members of an interconnected and less centralized team could be as fundamental to delivering higher quality care at a lower cost than reliance on medical technology, informational technology, and other infrastructural components [39]. It supports the value of “role-blurring” on multi-disciplinary teams [40], team structures that eschew the traditional physician centric medical model [41], and team-wide “flexible leadership” [42,43]. Our findings demonstrate that the Complex Care Clinic care coordination meetings enabled team members to strategize, brainstorm, problem solve, and critically reflect on how to better understand and care for high-utilizer patients. Flexible team leadership opened an inter-disciplinary communicative space to foster person-centred care-planning for complex patients. In particular, it allowed the group to move beyond the narrow focus of physical health and realize connections between the physical, emotional, and social components of patients’ lives and identify hidden factors undermining their progress. In fact, the explicit decision to not have team meetings led by physician members is consistent with what occurs in the highest functioning primary care clinics, where non-physician members have leadership roles, and when physicians do take the lead, they do so as servant leaders [44].

Incorporating these elements of team-based care enabled the Complex Care Clinic to effectively respond and adapt to patients’ complex biopsychosocial needs and unique challenges, barriers, preferences, and goals [45]. By integrating the clinical perspectives offered by the diverse team members, proactive and personalized strategies were created to prevent rapid deterioration of patients and unnecessary use of inappropriate, high cost health care services. These findings are supported by clinic results, including a 44% decline in inpatient admissions and a 38% decrease in emergency department use [23]. The relational foundation and personalized touch of these strategies increased patient engagement while also increasing empathy and understanding among Complex Care Clinic team members. This is especially important given the positive association between clinical empathy and improved health outcomes [46].

While interprofessional teamwork has already been recognized as a key component of effective care coordination in Complex Care Clinics, our findings underscore the importance of team diversity. We found social workers to be especially crucial, as their regular interactions with patients in non-clinical encounters allowed them to provide crucial insights into patients’ home and family lives. Conversely, while the social worker helped patients identify and navigate social services, his time and availability were limited. Incorporating other disciplines into Complex Care Clinic teams, such as occupational therapists and physical therapists, would complement the clinical and social services already offered by addressing many complex patients’ struggles with chronic pain, functional impairment, and limited mobility [47,48]. In addition, their prolonged engagement with patients during home-visits would render further insights into the complexity of patients’ lives.

Our intensive focus on a single Complex Care Clinic is the primary weakness of this study, as we were not able to isolate particular features of the program and evaluate their relative benefit to patient care or clinical outcomes. As such, our findings may be limited in generalizability beyond other safety-net settings with substantial numbers of uninsured, Medicaid, and dual Medicare-Medicaid eligible patients with socioeconomic and complex health challenges. Complex Care Clinics serving commercially insured patients with more stable social circumstances and fewer social needs may share a few common features but have unique differences. Furthermore, this study reflects the clinicians’ perspectives of the Complex Care Clinic and how they deliver care and does not include the patient’s perspective of receiving care. Additional research is needed to compare key features across Complex Care Clinics that serve a diverse array of patient populations.

## Conclusion and Recommendations

This “behind the scenes” understanding has important implications not only for existing primary care clinics, but for any setting that uses, or hopes to incorporate, team-based care. Moving away from a physician-led team to more flexible leadership can facilitate person-centered care of complex patients where social and emotional challenges impede physical health. While the wide array of team members in this study is atypical for the vast majority of primary care clinics, that is beginning to change as the Centers for Medicare and Medicaid Services is testing integrated behavioural health and primary care models. Health care teams will benefit from diverse team members such as registered nurse case managers, psychologists, and social workers – diverse interprofessional teams allow better connection of physical, emotional, and social components of health, can facilitate personalized strategies, and support better understanding of the “whole” patient by team members. Lastly, in order for the full benefit of team-based care to be realized, regular interprofessional communication and care planning among team members is essential. Our findings provide an important roadmap that team-based practices can follow as they develop models to most effectively meet the needs of their patient population.
